# Biodegradation tests of mercaptocarboxylic acids, their esters, related divalent sulfur compounds and mercaptans

**DOI:** 10.1007/s11356-018-1812-x

**Published:** 2018-04-17

**Authors:** Christoph Rücker, Waleed M. M. Mahmoud, Dirk Schwartz, Klaus Kümmerer

**Affiliations:** 1 Institute of Sustainable and Environmental Chemistry, Leuphana University Lüneburg, Universitätsallee 1, 21335 Lüneburg, Germany; 2 Analytical Chemistry Department, Faculty of Pharmacy, Suez Canal University, Ismailia, 41522 Egypt; 3 Bruno Bock Thiochemicals, 21436 Marschacht, Germany

**Keywords:** Ready biodegradability tests, Closed Bottle Test, Manometric Respirometry Test, Difunctional compounds, Mercaptocarboxylic acids, Mercaptocarboxylic acid esters, Mercaptans, Biodegradation rules of thumb

## Abstract

Mercaptocarboxylic acids and their esters, a class of difunctional compounds bearing both a mercapto and a carboxylic acid or ester functional group, are industrial chemicals of potential environmental concern. Biodegradation of such compounds was systematically investigated here, both by literature search and by experiments (Closed Bottle Test OECD 301D and Manometric Respirometry Test OECD 301F). These compounds were found either readily biodegradable or at least biodegradable to a significant extent. Some related compounds of divalent sulfur were tested for comparison (mercaptans, sulfides, disulfides). For the two relevant monofunctional compound classes, carboxylic acids/esters and mercaptans, literature data were compiled, and by comparison with structurally similar compounds without these functional groups, the influence of COOH/COOR’ and SH groups on biodegradability was evaluated. Thereby, an existing rule of thumb for biodegradation of carboxylic acids/esters was supported by experimental data, and a rule of thumb could be formulated for mercaptans. Concurrent to biodegradation, abiotic processes were observed in the experiments, rapid oxidative formation of disulfides (dimerisation of monomercaptans and cyclisation of dimercaptans) and hydrolysis of esters. Some problems that compromise the reproducibility of biodegradation test results were discussed.

**Electronic supplementary material:**

The online version of this article (10.1007/s11356-018-1812-x) contains supplementary material, which is available to authorized users.

## Introduction

Biodegradability in an aerobic aquatic phase is a key element in the environmental assessment of chemicals and is therefore generally considered in national and international chemicals regulations, e.g. the European Union REACH Regulation (EU 2006). One aim of such regulations is to minimise the release of persistent chemicals into the environment. While a persistent chemical is a potential threat to the environment, a chemical that is readily biodegraded to products such as CO_2_ and H_2_O (mineralisation) will not cause any harm to the environment.

Mercaptocarboxylic acids and their esters are a chemical class of potential concern, a particular class of difunctional compounds bearing both a mercapto (-SH) and a carboxylic acid or ester moiety (-COOH or -COOR’). Several members of this class are industrial chemicals, some are high production volume compounds of a global production capacity of several thousand metric tons per year. The major part of these products is used in an industrial setting, so that release to the environment can occur during production and industrial processing. However, there is also some use in consumer products, e.g. several thousand tons per year of thioglycolic acid and its salts go into cosmetic and cleaning formulations and are therefore released to the environment directly or indirectly via sewage treatment plants. It is therefore of interest to better understand the environmental impact of this class of chemicals, both of the parent compounds and of any transformation products. The present study should help to obtain a general view of these compounds’ biodegradation behaviour, by comparing literature data with our own experimental data to be generated.

For measuring biodegradability, a tiered approach is usually adopted in regulations, as described in guidance documents (OECD 1995, 2006; ECHA 2017). The first tier tests are screening tests described in the OECD technical guidance series 301A-F for ready biodegradability (OECD 1992). These tests are very stringent, offering unadapted microorganisms present in low concentration and diversity only limited opportunities for adaptation to and biodegradation of the chemical tested (test duration 28 days). They do not simulate real environmental conditions but give an indication of biodegradability of test compounds under various conditions (UN 2015).

A positive result in such a test (e.g. oxygen consumption 60% or more of theoretical oxygen demand within 28 days in tests 301D and 301F) can be considered as indication of rapid and more or less complete degradation under most environmental conditions including biological sewage treatment plants (STPs). Such a test result demonstrates conversion to stable inorganic products such as CO_2_, H_2_O, SO_4_^2−^ and NH_4_^+^ or NO_3_^−^ (OECD 2006; Guhl and Steber 2006). Chemicals passing an OECD 301 test are classified as readily biodegradable, and further testing is not normally required (OECD 2006; ECHA 2017). However, continuous release of such a substance may cause continuous exposure if degradation is slow compared to release, and further testing may therefore be envisaged (OECD 2006).

In contrast, a negative result in an OECD 301 test does not necessarily mean that the chemical will not be degraded under relevant environmental conditions, but it can be considered as indication of a potentially persistent chemical and may trigger second tier tests (inherent biodegradability, tests OECD 302A-C). If necessary, the highest (third) tier is applied, a simulation of biodegradation either in the aerobic treatment stage of STPs or in environmental compartments such as fresh or marine surface water (ECHA 2017; Kowalczyk et al. 2015). These higher tier tests require considerably more time, technical equipment, manpower and funds. Therefore, data from simulation tests are typically not available for most marketed chemicals, and biodegradability or persistence of a chemical in the environment is usually judged based on first or second tier tests only.

A compound’s biodegradability depends on its molecular structure and in particular on the functional groups present. Many compounds of environmental concern are complex, containing more than a single functional group, for example pesticides, herbicides, dyes and pharmaceuticals. Given the increasing number and complexity of chemicals and pharmaceuticals ending up in the aquatic environment, their biodegradability (or not so), decisive for their environmental impact, will increasingly influence environmental quality in the future. While some knowledge has accumulated on the biodegradation of monofunctional compound classes, the problem of biodegradation of difunctional or even more complex compounds was not adequately envisaged previously. As a consequence, some computer models for predicting the biodegradability of organic compounds are available, but they are built on information obtained largely from simple monofunctional compounds, and their predictive ability for multifunctional compounds is therefore questionable (Rücker and Kümmerer 2012). For example, models such as the Biowin models (US EPA) generally assume additive effects of various structural fragments, though non-additive interactions of functional groups are to be expected. A goal to be aimed at in the future is prediction of any compound’s biodegradation behaviour from its molecular structure, including complex structures. This will also allow design of environmentally biodegradable chemicals. All this requires a large body of data for building and validating models, data that are largely not available hitherto. In view of all combinations of functional groups that may be present in an environmental contaminant, there is a huge amount of work to be done, and our study on mercaptocarboxylic acids is thus a first step in a direction that needs to be pursued for several other classes of difunctional compounds.

Understanding the behaviour of difunctional compounds obviously requires as a prerequisite thorough knowledge of how the single functional groups involved influence the respective outcome. It is now well known that some molecular substructures enhance or hinder biodegradation. These findings were formulated as “rules of thumb” that are based on more or less evidence. Relevant in our context, many carboxylic acids and esters are rather well biodegradable. So the rule “carboxylic acid or ester groups enhance biodegradation” became popular (Howard 2000; Boethling et al. 2007; Cheng et al. 2012). However, it turned out that data supporting this rule were never explicitly compiled, nor was it specified against which compound a carboxylic acid or ester was to be compared with respect to biodegradability, the compound bearing a methyl group or the one bearing a H atom in place of the COOH/COOR’ group.

For the other monofunctional compound class of interest here, mercaptans, some biodegradability data are available but a rule of thumb was not formulated hitherto.

Thus, questions to be addressed in the present study were the following.

1. Is the rule of thumb for biodegradability of carboxylic acids/esters well based on experimental data?
2. Can a rule of thumb be formulated for biodegradability of mercaptans, based on experimental data?
3. What is the biodegradation behaviour of compounds containing both a carboxylic acid/ester and a mercaptan functional group? Can a general rule be given on the biodegradability of this class of difunctional compounds?

We therefore first searched the literature for biodegradation of carboxylic acids and esters and corresponding compounds without these functional groups, in order to confirm or refute the mentioned rule of thumb. In a second step, the literature was searched for biodegradation of mercaptans and corresponding hydrocarbons and analogous alcohols, in order to obtain a rule of thumb for biodegradation of mercaptans. As to the third question, available experimental biodegradation data for mercaptocarboxylic acids and esters turned out to be partially contradictive, which may be explained by their origin from tests performed according to various protocols in various laboratories. We therefore decided to measure biodegradation of several mercaptocarboxylic acids and esters and, for comparison, of several miscellaneous divalent sulfur compounds (mercaptans, sulfides, disulfides) in two standard ready biodegradability tests, the Closed Bottle Test (CBT, 301D) and the Manometric Respirometry Test (MRT, 301F), using a single source of inoculum. Thereby, we expected to obtain more consistent results. In addition to the OECD 301D and F methodology, LC-UV/MS/MS was employed to identify any transformation products.

## Experimental

Acetonitrile and methanol (HiPerSolv CHROMANORM, LC-MS grade, BDH Prolabo) and formic acid (analytical grade) were purchased from VWR International GmbH (Darmstadt, Germany). Dithiodiglycolic acid (CAS-RN 505-73-7, DTDGA, TGA disulfide) and 3,3′-dithiodipropionic acid (CAS-RN 1119-62-6, DTDPA, 3-MPA disulfide) were obtained from Sigma-Aldrich (Steinheim, Germany), dimethyl 3,3′-dithiodipropionate (CAS-RN 15441-06-2, MMP disulfide) from abcr GmbH, Karlsruhe, Germany. 1,2,5-Trithiepane (CAS-RN 6576-93-8) was purchased from Envilytics, Wiesbaden, Germany. All other tested organosulfur compounds were provided by Bruno Bock Thiochemicals (Marschacht, Germany), were of technical grade and were used without purification. Some of these are multiconstituent substances (MCSs): isotridecyl thioglycolate (CAS-RN 57417-85-3, iC_13_TG), isotridecyl 3-mercaptopropionate (CAS-RN 1040871-35-9, iC_13_MP), isooctyl thioglycolate (CAS-RN 25103-09-7, iOTG) and isooctyl 3-mercaptopropionate (CAS-RN 30374-01-7, iOMP) are mixtures of esters obtained from the respective carboxylic acid (thioglycolic acid or 3-mercaptopropionic acid) and mixtures of branched mostly C_13_ or C_8_ primary alcohols (CAS-RN 68526-86-3 and 68526-83-0, respectively).

Trimethylolpropane tris(3-mercaptopropionate) (CAS-RN 33007-83-9, TMPMP), pentaerythrityl tetrakis(3-mercaptopropionate) (CAS-RN 7575-23-7, PETMP), dipentaerythrityl hexakis(3-mercaptopropionate) (CAS-RN 25359-71-1, DiPETMP) and ethoxylated trimethylolpropane tris(3-mercaptopropionate) (CAS-RN 345352-19-4, 674786-83-5, ETTMP 700) are mixtures of esters obtained from an excess of 3-mercaptopropionic acid and the respective multifunctional alcohol (trimethylolpropane, pentaerythritol, dipentaerythritol, ethoxylated trimethylolpropane). Along with the major constituent, they contain partially esterified products as well as thiolesters.

Myristyl thioglycolate (CAS-RN 84238-40-4, C_14_TG) is a mixture of mostly non-branched C_10-16_ alkyl esters of thioglycolic acid.

Glyceryl monothioglycolate (CAS-RN 30618-84-9, GMT) is a reaction mixture of glycerol and thioglycolic acid. Main constituents are the isomeric monoesters, minor constituents are di- and triesters, as well as free glycerol and thioglycolic acid.

The mineral medium used in the experiments was prepared according to the OECD 301 guidelines from deionised water (Miele Aqua Purification model G 7795, conductivity ≤ 5 μS/cm) and analytical grade reagent salts in the concentrations specified there (OECD 1992). Effluent of the municipal sewage treatment plant (STP) of Lüneburg, Germany (144,000 inhabitant equivalents) was collected on the day of test start. The effluent sample was filtered and then used as inoculum directly. The Lüneburg STP treats typical municipal sewage, there is no industry connected to the STP that deals with the compounds studied here. As to the presence of other organic or toxic substances, controls were run according to the guidelines: the quality control results and the inoculum blank results were as required (validity criteria number 2 and 4 below).

The Closed Bottle Test (CBT, OECD 301D) is considered the most stringent among the OECD 301 series ready biodegradability (RB) tests (OECD 1992). It works at low test compound concentration (theoretical oxygen demand (ThOD) ~ 5 mg/L) and low bacterial density (10^4^–10^6^ colony forming units (CFU)/mL). In our CBT modification, we used as inoculum two drops of STP effluent per litre of mineral solution. This inoculum amount was enough for degrading sodium acetate sufficiently (validity criterion 2, see below), at the same time being safe with respect to criterion 4.

The test comprised four completely filled (no headspace) bottles (inoculum blank, quality control, test proper, toxicity control, each in duplicate) and was run for 28 days at 20 ± 1 °C in the dark (OECD 1992). For details, see Table S1 in the Electronic supplementary material.

Oxygen concentration in the bottles was monitored daily from outside using the Fibox3 system (fiber-optic oxygen meter with temperature sensor, Precision Sensing GmbH, Regensburg, Germany) based on a sensor spot in each bottle (Friedrich et al. 2013). The temperature was monitored daily; the pH was measured at days 0 and 28, was adjusted to 6.5–8 if necessary at day 0 and was in this range at day 28.

For a CBT result to be valid, five criteria must be met simultaneously:

1. The difference in degradation between replicate test bottles should be less than 20%.^1^
2. The biodegradation of reference compound (sodium acetate) in the quality control has to be at least 60% by day 14.
3. The biodegradation in the toxicity control should be at least 25% of the total ThOD by day 14.
4. Oxygen consumption in the inoculum blank should not exceed 1.5 mg/L by day 28.
5. Oxygen concentration in the test bottles should not fall below 0.5 mg/L at any time.

The Manometric Respirometry Test (MRT, OECD 301F) uses a higher test compound concentration (50–100 mg ThOD/L according to the guideline, ~ 30 mg ThOD/L in our modification) and higher bacterial density (10^7^–10^8^ CFU/mL according to the guideline, 80 mL STP effluent/L final solution in our work) and thus higher bacterial diversity (OECD 1992). Another difference between CBT and MRT is the implementation of a further control bottle (sterile control) and the use of only one bottle for toxicity control. For details, see Table S1 in the Electronic supplementary material. The test was run for 28 days at 20 ± 1 °C in the dark with gentle stirring. Oxygen consumption was recorded daily using an OxiTop control OC110 system (WTW, Weilheim, Germany), measuring the pressure decrease in the headspace (about one third of the bottle volume), while CO_2_ produced was removed by adsorption/absorption to NaOH pellets/conc. NaOH solution. The temperature was monitored daily, the pH was measured at days 0 and 28, was adjusted to 6.5–8 if necessary at day 0 and was in this range at day 28.

For a MRT result to be valid, five criteria must be met simultaneously:

- 1.–3. The first three criteria are the same as in CBT.
- 4. The oxygen consumption in the inoculum blank should be at most 60 mg/L by day 28.
- 5. If the oxygen consumption by the test substance at test end is < 60%, then the pH must be within the range 6–8.5.

The pass level in both CBT and MRT is oxygen consumption at day 28 of 60% of the theoretical oxygen demand. ThOD was calculated under the assumption that sulfur is oxidised to SO_4_^2−^, according to the guideline (OECD 1992). To be considered readily biodegradable, a substance is further required to achieve the pass level within 10 days after the first 10% degradation occurred (“10 day window”), except for the 301C test.

Sufficiently soluble compounds were dissolved in mineral solution and then dispensed into the bottles, whereas compounds of insufficient solubility were, separately for each bottle, weighed using a miniature polystyrene weighing boat, thrown into the bottle and then suspended in mineral solution (“direct weighing”). In both tests at test begin (day 0) and test end (day 28), samples were taken (except in cases of direct weighing) and were stored at − 80 °C until analysis. Samples were analysed for the test compound (primary elimination) and for transformation products (TPs) by HPLC-UV and HPLC-UV-MS/MS (ion trap) whenever possible. In most cases, MRT samples rather than CBT samples were analysed since the higher concentration facilitated identification of TPs.

For HPLC-UV, a Prominence HPLC instrument (Shimadzu, Duisburg, Germany) was used. Separation was performed on a RP-18 column (NUCLEODUR^®^ 100-3 C18 ec, 2 mm ID, 125 mm, Macherey & Nagel, Düren, Germany) protected by an EC guard column (NUCLEODUR^®^ 100-3 C18 ec, 4 × 2 mm). Elution was isocratic or gradient using mixtures of 0.1% formic acid in water (solution A) and 100% acetonitrile (solution B). The flow rate was 0.25 mL/min, the oven temperature 40 °C and the detector wavelength 210 nm. Twenty to fifty microlitres of each sample were injected without any workup.

HPLC-UV-MS was performed on an Agilent Technologies series 1100 HPLC instrument (Agilent Technologies, Böblingen, Germany). Column, eluent solutions and run parameters were as above. To the chromatograph, a Bruker Daltonic Esquire 6000 plus ion-trap mass spectrometer was coupled, equipped with an atmospheric pressure electrospray ionisation (ESI) interface and a Bruker data analysis system (Bruker Daltonic GmbH, Bremen, Germany). The mass spectrometer was operated in the positive mode. For analytical details, see the “Electronic supplementary material”.

## Results and discussion

### Biodegradation data from the literature

Tables 1 and 2 show biodegradation test results published in the scientific and regulatory literature. The purpose of Tables 1 and 2 is to provide a semi-quantitative overview of the compounds’ biodegradability as a function of specific functional groups present in the molecules. These data come from different tests and therefore are not strictly comparable, they should not be used indiscriminately together, e.g. for training of a biodegradation QSAR model.

**Table 1:**
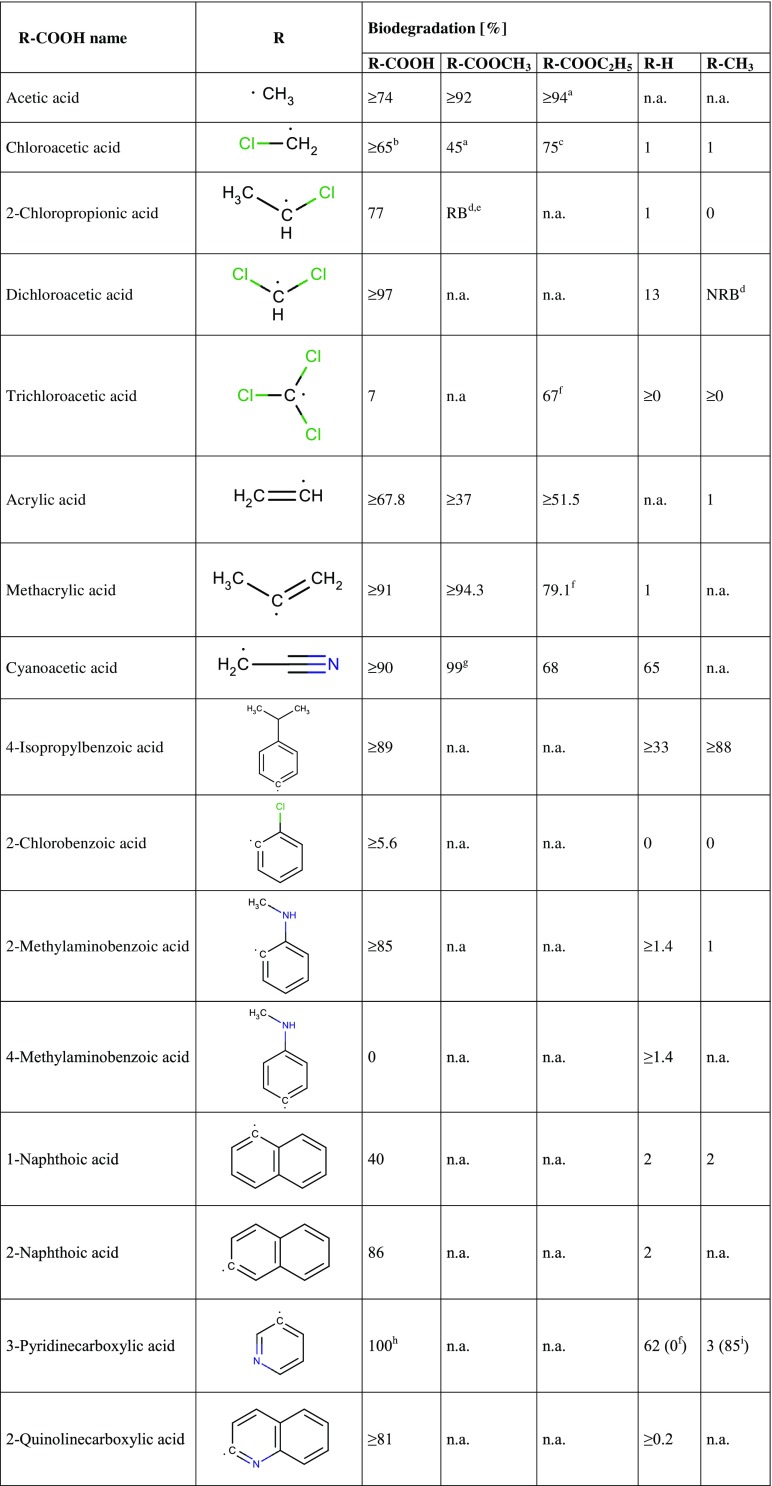
Biodegradation test results (oxygen consumption as % of the theoretical oxygen demand) of carboxylic acids, esters and corresponding compounds without these functional groups, taken from the literature. Data are primarily from the 28-day (or 14-day) OECD 301C (MITI-I) test

*n.a.* not available, ^a^The corresponding carboxylic acid was formed to some extent during the test

^b^Test duration 21 days, ^c^OECD 301F, ECHA database, ^d^Test unspecified, Cheng et al. (2012), ^e^Test unspecified, Pizzo et al. (2013)

^f^OECD 301D, ECHA database, ^g^OECD 301A, ECHA database, ^h^OECD 301E, ECHA database, ^i^OECD 301B, ECHA database

**Table 2:**
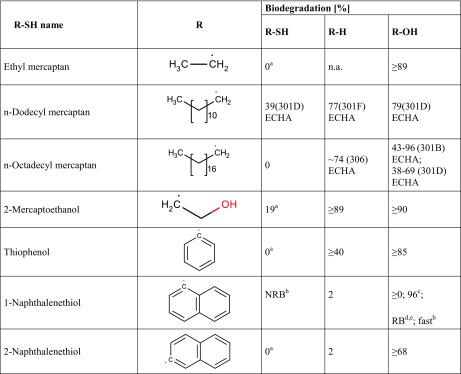
Biodegradation results (oxygen consumption as % of the theoretical oxygen demand) of mercaptans and corresponding hydrocarbons and alcohols, taken from the literature. Data are primarily from the 28-day (or 14-day) OECD 301C (MITI-I) test

*n.a.* not available

^a^The corresponding disulfide was formed to some extent during the test

^b^Test unspecified, Biowin1/2 training set

^c^Test unspecified, Pizzo et al. (2013)

^d^Test unspecified, Cheng et al. (2012)

^e^Biowin5/6 training set (MITI-I model)

Unless stated otherwise, results in Tables 1 and 2 were obtained in the 28-day (or 14-day) OECD 301C (MITI-I) test, as contained in the J-CHECK database. Results of the 14-day test are given as “≥xx”, where xx is the numerical result measured after 14 days. In cases of no result found in the J-CHECK database, the REAXYS and ECHA databases were consulted, then training and test datasets of the Biowin1/2 and Biowin5/6 models (US EPA EPI Suite), and as a last resort experimental data cited in recent papers on biodegradation modelling (Cheng et al. 2012; Pizzo et al. 2013). The latter sources give results as readily biodegradable/not readily biodegradable (RB/NRB) only.

#### Carboxylic acids and their esters

In Table 1, biodegradation test results are compiled for carboxylic acids and their methyl or ethyl esters and for the corresponding methyl compound and the compound lacking that methyl group. Each row of Table 1 contains data for (at least) a pair of compounds, so that comparison within a row shows the impact of a COOH/COOCH_3_/COOC_2_H_5_ group on biodegradability in context R. Comparison between rows then shows whether the effect is same or different in different structural contexts as described by the variation of R.

As an exception, we included acetic acid despite the lack of data for methane and ethane, for the relevance of sodium acetate as the reference compound in biodegradability tests.

Examination of Table 1 shows that a carboxylic acid or its methyl or ethyl ester is more easily biodegradable than corresponding compounds bearing a CH_3_ or H at the same position, and this seems to be generally true. For the pair 4-methylaminobenzoic acid and *N*-methylaniline, seemingly a counterexample, the difference between 0 and 1.4% is within experimental uncertainty. Thus, the rule of thumb for carboxylic acids/esters seems to be well based on facts.

No general trend in biodegradability is seen in Table 1 on comparing the esters to the carboxylic acids.

Partial hydrolysis of esters to alcohols and acids is to be expected in aqueous solution even at neutral pH and 20 °C during a reaction time extended as long as 28 days and was in fact observed for several esters in the OECD 301C test. Examples are ethyl acetate and methyl chloroacetate in Table 1, and propyl acetate, *sec*-butyl acetate, *n*-butyl acrylate, methyl 4,4-dimethyl-3-oxopentanoate, acetylsalicylic acid (J-CHECK database). Hydrolysis is a step neutral with respect to oxidation, it does not show up in the oxygen balance but may influence the biodegradation rate by producing more water-soluble and bioavailable species.

#### Mercaptans

Table 2 presents biodegradation test results of mercaptans and the corresponding hydrocarbons and alcohols for comparison. Each row of Table 2 contains data for (at least) a pair of compounds, so that comparison within a row shows the impact of a SH (or OH) group on biodegradability in structural context R. Comparison between rows then shows whether the effect is same or different in different structural contexts as described by the variation of R.

The limited data in Table 2 show that a mercapto group is detrimental to biodegradation compared with the corresponding hydrocarbon, in contrast to a hydroxyl group that enhances biodegradation. This trend is seen in all rows of Table 2 and so may be considered a rule of thumb.

A mercapto group, however, does not absolutely prohibit biodegradation, as demonstrated by *n*-dodecyl mercaptan and 2-mercaptoethanol. This latter result may be interpreted as being caused by the presence of structural elements both favouring (hydrocarbon chain of intermediate length, OH group) and hindering biodegradation (SH).

The data for 1-naphthol demonstrate that published experimental biodegradation test results are generally to be considered with caution: the 14-day MITI-I test resulted in 0% biodegradation, whereas the training set of models Biowin5/6 claims a MITI-I test result of > 60% (“readily biodegradable”), as do Cheng et al. (2012) for an unspecified test. Pizzo et al. (2013) even report 96% biodegradation in an unspecified test, and the Biowin1/2 training set claims “fast biodegradation” for this compound. The Biowin1/2 data of 1-naphthol is from the Environmental Fate Database of Syracuse Research Corporation, which unfortunately is no longer publicly available. Further, the four “high” claims may result from tapping the same source rather than coming from four independent experiments.

### Experimental biodegradation data from the present study

We tested biodegradation of 24 substances containing divalent sulfur, i.e. mercaptocarboxylic acids, their esters, disulfides, sulfides and mercaptans. As a first experimental result of the present study, for all tested compounds in all biodegradation experiments no indication of toxicity to the bacteria was found at the tested substance concentrations.

Table 3 presents the biodegradation test results obtained in this study. In case of an invalid result, the test was repeated until a valid result was obtained. For a few compounds, a test was repeated despite of a valid first result already obtained, in order to examine intra-laboratory reproducibility. For all results ≥ 60%, the 10-day window was passed.

**Table 3:** Biodegradation test results obtained in the present study. For each experiment, the % O_2_ consumption in the two parallel bottles is given, and for valid results the average ±SD, otherwise the criterion rendering a result invalid. For structures, see Table 7

Compound name (abbreviation) [CAS-RN]	Experiment number	OECD 301D (CBT)		OECD 301F (MRT)	
		O_2_ consumption/ThOD [%]	Average in a valid experiment ±SD	O_2_ consumption/ThOD [%]	Average in a valid experiment ±SD
**TGA and derivatives**					
Thioglycolic acid (TGA) [68-11-1]	1	31.8, 45.3	38.5 ± 9.5	114.4, 92.1	Not valid, criterion 1
	2	49.2, 51.3	50.2 ± 1.5	78.8, 94.4	86.6 ± 11.1
Diammonium dithiodiglycolate (DADTDG, TGA disulfide ammonium salt) [68223-93-8]	1 calculated without nitrification	123.8, 106.4	115.1 ± 12.3	130.0, 132.0	131.0 ± 1.4
	1 re-calculated with nitrification	76.6, 65.9	71.3 ± 7.6	80.5, 81.7	81.1 ± 0.9
	2 calc. with nitrification	14.9, 29.2	22.0 ± 10.1	79.4, 90.8	85.1 ± 8.0
	3 calc. with nitrification	90.9, 86.0	88.5 ± 3.5		
Dithiodiglycolic acid (DTDGA, TGA disulfide) [505-73-7]	1	65.9, 62.2	64.1 ± 2.6	87.9, 111.2	Not valid, criterion 1
	2			60.3, 55.7	58.0 ± 3.3
2-Ethylhexyl thioglycolate (2-EHTG) [7659-86-1]	1*	58.3, 61.2	59.7 ± 2.0	28.2, 54.7	Not valid, criterion 1
	2*			18.4, 13.9	16.2 ± 3.2
Isotridecyl thioglycolate (iC_13_TG)^a^ [57417-85-3]	1**	1.4, 10.0	Not valid, criterion 4	40.9, 22.5	31.7 ± 13.0
	2**	14.6, 9.5	12.0 ± 3.6		
Glycol di(mercaptoacetate) (GDMA) [123-81-9]	1	62.2^b^	Not valid, criterion 1	62.1, 69.8	65.9 ± 5.4
	2	82.5, 72.4	Not valid, criterion 4		
	3	70.0^b^	Not valid, criterion 1		
	4	79.4, 77.1	78.3 ± 1.6		
Glyceryl monothioglycolate^a^ (GMT) [30618-84-9]	1	56.0, 50.7	53.4 ± 3.7	79.8, 80.8	80.3 ± 0.7
**3-MPA and derivatives**					
3-Mercaptopropionic acid (3-MPA) [107-96-0]	1	0.4, 32.4	Not valid, criterion 1	78.7, 87.0	82.8 ± 5.9
	2	27.4, − 7.3	Not valid, criteria 1 and 4	101.4, 90.1	95.8 ± 8.0
	3	0.6, 3.8	2.2 ± 2.3		
Dithiodipropionic acid (DTDPA, 3-MPA disulfide) [1119-62-6]	1	3.9, 1.5	2.7 ± 1.7	121.6, 87.9	Not valid, criterion 1
	2			93.7, 100.3	97.0 ± 4.7
Methyl 3-mercaptopropionate (MMP) [2935-90-2]	1	65.0, 61.7	63.4 ± 2.3	23.3, 12.0	17.7 ± 8.0
	2	16.0, 21.0	18.5 ± 3.6		
	3	74.1, 14.1	Not valid, criterion 1		
	4	5.4, 12.3	8.8 ± 4.9		
Propanoic acid, 3,3′-dithiobis-, dimethyl ester (MMP disulfide) [15441-06-2]	1	6.6, 4.1	5.4 ± 1.8	61.6, 84.2	Not valid, criterion 1
	2	10.6, 13.0	11.8 ± 1.7	14.0, 21.7	17.8 ± 5.4
	3*			21.6, 1.3	Not valid, criterion 1
Butyl 3-mercaptopropionate (BuMP) [16215-21-7]	1	55.6, 29.0	Not valid, criterion 1	15.0, 20.7	17.8 ± 4.0
	2	57.4, 60.3	Not valid, criterion 4		
	3	61.9, 62.5	62.2 ± 0.5		
	4	40.8, 41.1	41.0 ± 0.2		
2-Ethylhexyl 3-mercaptopropionate (2-EHMP) [50448-95-8]	1*	54.3, 49.8	52.1 ± 3.2	56.4, 27.0	Not valid, criterion 1
	2*			42.4, 51.6	47.0 ± 6.5
Isooctyl 3-mercaptopropionate (iOMP) [30374-01-7]	1**	31.1, 32.1	31.6 ± 0.8	− 8.2, 4.6	− 1.8 ± 9.0
Isotridecyl 3-mercaptopropionate (iC_13_MP)^a^ [1040871-35-9]	1**	10.5, 17.6	14.1 ± 5.0	29.5, − 11.1	Not valid, criterion 1
	2**			34.2, 35.8	35.0 ± 1.2
Glycol di(3-mercaptopropionate) (GDMP) [22504-50-3]	1	2.1, − 0.8	0.7 ± 2.0	73.4, 39.8	Not valid, criterion 1
	2	10.4, 13.6	12.0 ± 2.3	32.8, 50.4	41.6 ± 12.5
	3*			33.4, 41.0	37.2 ± 5.3
Trimethylolpropane tris-(3-mercaptopropionate)^a^ (TMPMP) [33007-83-9]	1**	8.2, 9.9	9.1 ± 1.2	32.9, 55.4	Not valid, criterion 1
	2**			4.7, 1.7	3.2 ± 2.1
Dipentaerythrityl hexakis-(3-mercaptopropionate)^a^ (DiPETMP) [25359-71-1]	1**	1.4, 3.3	2.4 ± 1.4	14.8, 42.4	Not valid, criterion 1
	2**			12.0, − 5.5	3.2 ± 12.3
Ethoxylated trimethylolpropane tris-(3-mercaptopropionate)^a^ (ETTMP 700) [674786-83-5 or 345352-19-4]	1**	3.1, − 3.2	Not valid, criterion 4	35.1, 40.0	37.6 ± 3.5
	2**	9.2, 8.6	8.9 ± 0.5		
**Miscellaneous compounds containing divalent sulfur**					
Thiolactic acid (TLA) [79-42-5]	1	11.9, 12.8	12.4 ± 0.6	66.6, 75.9	71.2 ± 6.6
Di(2-ethylhexyl) thiodiglycolate(Di-2-EHTDG) [24293-43-4]	1**	19.3, 14.0	Not valid, criterion 4	41.5, 34.3	37.9 ± 5.1
	2**	22.7, 33.8	28.3 ± 7.9		
Methylene bis(butyl thioglycolate) (MBT) [14338-82-0]	1*	49.4, 48.5	48.9 ± 0.7	56.6, 63.1	59.8 ± 4.6
	2*			52.2, 63.2	57.7 ± 7.8
Bis(2-mercaptoethyl) sulfide (DMDS) [3570-55-6]	1	− 0.2, − 2.0	− 1.1 ± 1.3	35.8, − 12.9	Not valid, criterion 1
	2*			1.8, 33.4	Not valid, criterion 1
4-Mercaptomethyl-3,6-dithia-1,8-octanedithiol (DMPT) [131538-00-6]	1	− 5.0, − 2.7	− 3.9 ± 1.6		

^a^Multiconstituent substance (MCS), for details, see “Experimental”

^b^Result from one test bottle only, the other one became leaky during the experiment

*Due to poor solubility, this substance was directly weighed in MRT

**Due to very poor solubility, this substance was directly weighed both in CBT and in MRT

Tested substances are subdivided in Table 3 into three groups. Group 1 contains thioglycolic acid (TGA) and its derivatives (ammonium salt, disulfide and esters). Group 2 comprises 3-mercaptopropionic acid (3-MPA) and its derivatives (disulfides and esters). Group 3 contains miscellaneous compounds of divalent sulfur, e.g. thiolactic acid (TLA), thioethers (MBT, Di-2-EHTDG) and mercaptans (DMDS, DMPT).

### Discussion of reproducibility

Generally, reproducibility is an issue in all biodegradability test results. A closer look on our data reveals that poor reproducibility appears on various levels, as detailed in the following subsections.

#### Intra-experiment reproducibility

Table 4 gathers discrepancies between duplicate bottles within an experiment from Table 3 for CBT and MRT, i.e. all those results that are invalid for violation of criterion 1.

**Table 4:** Non-reproducibility within the same experiment

CBT biodegradation [%]		MRT biodegradation [%]	
3-MPA	0, 32; − 7, 27	TGA	92, 114
MMP	14, 74	DTDGA	88, 111
BuMP	29, 56	2-EHTG	28, 55
		3-MPA disulfide	88, 122
		MMP disulfide	1, 22; 62, 84
		2-EHMP	27, 56
		iC_13_MP	− 11, 30
		GDMP	40, 73
		TMPMP	33, 55
		DiPETMP	15, 42
		DMDS	− 13, 36; 2, 33

Discrepancies between duplicate bottles in the same experiment were occasionally observed earlier (Painter and King 1985), the guidelines explain this phenomenon by differences in the composition of the inoculum (ECHA 2017, page 214; OECD 1992). Nevertheless, some authors consider such results not only “invalid”, but also useless, meaning that “something went wrong with the experiment”. The experiment is then repeated until a valid result is obtained, and invalid results are discarded rather than reported. This practice leads to the wrong impression that biodegradation experiments are as reproducible as physicochemical measurements. To correct this view, we here report our invalid experiments also and offer alternative explanations. Moreover, such invalid experimental results are by no means useless, in particular if both parallel bottles exceed the pass level, as is the case for TGA, DTDGA, 3-MPA disulfide and MMP disulfide in MRT (Table 4).

At least some of the discrepancies observed in the present experiments may be explained by experimental problems:

- Poorly soluble substances (2-EHMP, 2-EHTG, iC_13_MP, TMPMP, DiPETMP, MMP disulfide in its experiment 3) were directly weighed into the test bottles, suffering from difficulties in weighing mg amounts of viscous material into several parallel bottles.
- GDMP (in MRT experiment 1) and MMP disulfide (in MRT experiment 1) were tested applying the usual substance preparation as a solution despite borderline solubility. Incomplete solution then may have resulted in different amounts of test substance in the two parallel bottles.
- In two CBT experiments with GDMA, one of the two parallel bottles became leaky during the test, as manifested by the O_2_ concentration in one bottle unexpectedly increasing after some days of decrease. These bottles were therefore excluded from further consideration. However, a miniature leak will have a less obvious effect, the oxygen concentration decreasing merely more slowly than in a tight bottle, so that the apparent oxygen consumption in that bottle will lag behind. This is what we observed repeatedly in this study. Thereby a final difference in apparent biodegradation of > 20% may or may not build up between parallel bottles. Thus, a possible tiny leak is a sufficient explanation for any discrepancies between parallel bottles at day 28.
- Finally, bacterial density and diversity may differ in parallel bottles following from uneven dispersion of bacterial species in the mineral solution. If, e.g. competent bacteria form aggregates, then two samples (same volume) taken from a highly dilute suspension may contain different numbers of cells of different diversity just by chance.

#### Intra-laboratory reproducibility

Few scientists perform a second or third biodegradation experiment if a valid result is already at hand. If such double measurements are nevertheless done and produce conflicting results, each being valid, then such data likely will not be published. Therefore, this kind of problem also is often not apparent, it is not explicitly mentioned in the guidelines, and its prevalence is unknown.

Table 5 gathers discrepancies from Table 3 between different experiments performed for the same compound under the same test protocol in the present study, from CBT and MRT.

**Table 5:** Reproducibility of test results, valid results only, i.e. each number is the mean from two bottles differing by no more than 20%

CBT biodegradation [%]		MRT biodegradation [%]	
DADTDG	22, 71, 89	DADTDG	81, 85
GDMP	1, 12	GDMP	37, 42
TGA	39, 50	3-MPA	83, 96
MMP	9, 18, 63	MBT	58, 60
MMP disulfide	5, 12		
BuMP	41, 62		

In our experiments, MRT results seem to be more reproducible than CBT results (Table 5). The CBT results for three out of six compounds led to conflicting assessments (above and below the pass level), while in MRT this happened for one out of four compounds only. This difference can be explained by the higher bacterial density/diversity in MRT compared to CBT.

Discrepancies between two seemingly identical biodegradation experiments performed at different times may be due to different lag times. Lag time is the time required for adaptation by the microbial population to metabolise the substrate efficiently. Different lag times can be caused by a variation in the diversity of the bacterial population in two experiments, that is, by presence or absence of competent bacteria. In a former study, bacterial strains were isolated from activated sludge that not only tolerate organosulfur carboxylic acid esters, but even were able to use them as sole carbon and energy source (Toups et al. 2010). Such specialist strains are not necessarily those that biodegrade these compounds in aerobic real environments or activated sludge, since they may be outcompeted by generalists. However, if sufficient amounts of these compounds are available as sole carbon source as in an OECD 301 experiment, such specialist strains tend to increase due to induction of appropriate catabolic enzymes.

**Fig. 1:**
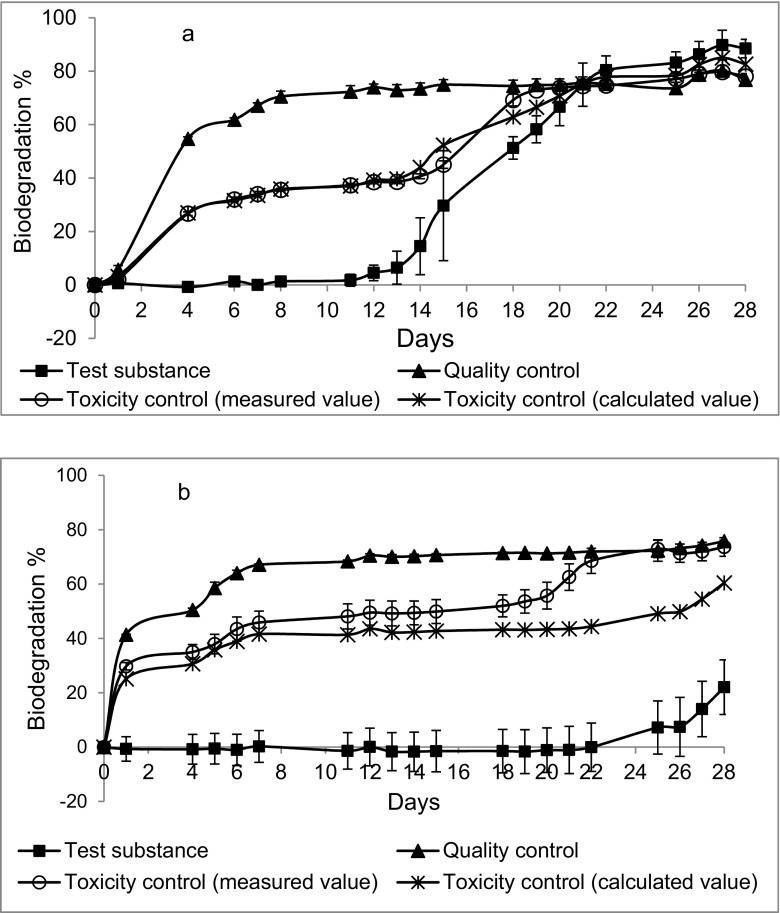
Biodegradation curves of DADTDG in two different CBT experiments. **a** Experiment 3, lag phase 14 days. **b** Experiment 2, lag phase 27 days

If the sigmoid biodegradation curve is shifted more or less along the time axis, it hits the fixed 28-day terminus at different points on the biodegradation axis. A shift of a few days can make the difference between very low and very high biodegradation. As an example, consider the biodegradation curves of DADTDG, the ammonium salt of TGA disulfide (Fig. 1). DADTDG was readily biodegraded (two valid CBT results, 71 and 89%, and two valid MRT results, 81 and 85%), but one valid CBT result of 22% biodegradation was also obtained (experiment 2). Unusually long and variable lag phases were observed in CBT (19 days in experiment 1, resulting in 71% biodegradation; 27 days in experiment 2, 22%; 14 days in experiment 3, 89%). On the other hand, in MRT lag times were in good concordance (9 and 10 days), as were the degrees of biodegradation.

Bacterial populations taken from the same source at different times are not alike, but are subjected, e.g., to seasonal variations (Brillet et al. 2016). In particular, the amount of rare microbial specialists in activated sludge of a sewage treatment plant (STP) was found to show considerable temporal (seasonal) variation (Kim et al. 2013; Ju et al. 2014). In our case, the inoculum for each experiment was freshly taken from the same source, effluent of the Lüneburg municipal STP, but the delay between subsequent experiments with the same substance was between 2 weeks and 9 months, enough time for seasonal variation of the bacteria population in the STP.

#### Variation between test results for the same compound under different OECD 301 test protocols

For many substances, Table 3 shows strong differences between CBT (OECD 301D) and MRT (OECD 301F) results. Though CBT is meant to be the most stringent test, in some instances, degradation in CBT was higher than in MRT. Such a phenomenon has been known for many years, and recent comparisons between 301C and 301X test results based on a large experimental basis revealed that for some compounds, 301C was more efficient, for others, 301X for each X from {A, B, D, E, F} (Kayashima et al. 2014).

#### Differences between structurally similar compounds in the same test

Ideally, it is expected that structurally similar compounds should behave similarly under the same 301 test protocol. Table 6 compiles the experimental data (from Table 3) of structurally similar compounds in the same test.

**Table 6:** Results for structurally similar compounds in the same test (valid results only)

Chemical class	Compound	CBT biodegradation [%]	MRT biodegradation [%]
Mercaptocarboxylic acids	3-MPA	2	83, 96
	TGA	39, 50	87
	TLA (2-MPA)	12	71
Dithiodicarboxylic acids	DTDPA	3	97
	DTDGA	64	58
Ethylhexyl esters	2-EHMP	52	47
	2-EHTG	60	16
Glycol esters	GDMP	1, 12	37, 42
	GDMA	78	66
Isotridecyl esters	iC_13_MP	14	35
	iC_13_TG	12	32
Mercaptocarboxylic acids/esters and corresponding disulfides	3-MPA	2	83, 96
	3-MPA disulfide	3	97
	MMP	9, 18, 63	18
	MMP disulfide	5, 12	18
	TGA	39, 50	87
	DTDGA	64	58

As Table 6 shows, some pairs of similar compounds more or less meet the expectation (3-MPA, TGA, TLA; iC_13_MP, iC_13_TG; 3-MPA, 3-MPA disulfide; MMP, MMP disulfide), while others do not. In case of a mercaptan and its disulfide, concordance is explained by a test of the former being essentially a test of the latter, see “Primary elimination and transformation products”. Table 6 suggests not to overestimate both concordant and divergent behaviour of similar compounds in single biodegradation experiments, given all the experimental imponderability discussed above. It is probably naive to expect similar compounds to behave similarly in a single biodegradation experiment when even repeated experiments on the same compound can lead to contradictions. We have to realise that a biodegradation test result for a compound is only one of several possibilities, in other words that we should consider biodegradation experiments as statistical processes in the sense that they are governed by some factors difficult to control. Similarity in the behaviour of similar compounds can then be expected only on the basis of several or even many experiments. Even then, the often high sensitivity of enzymes to small structural differences of potential substrates may lead to unexpected effects within a series of related structures.

### Synopsis of biodegradation data

Table 7 is a synopsis of biodegradation test results for mercaptocarboxylic acids and esters and miscellaneous divalent sulfur compounds. Along with the 24 substances from Table 3, Table 7 contains seven further compounds with available biodegradation results that were not included in the present experimental study.

**Table 7:**
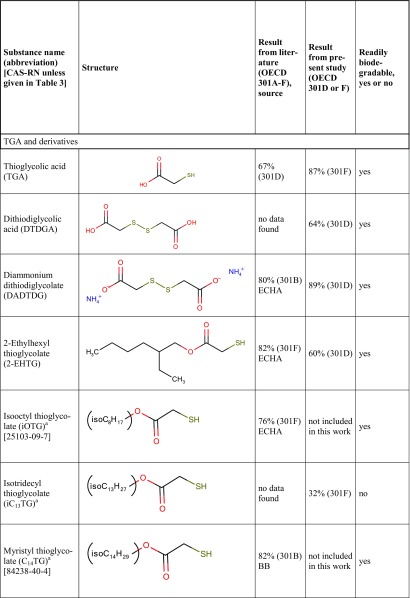

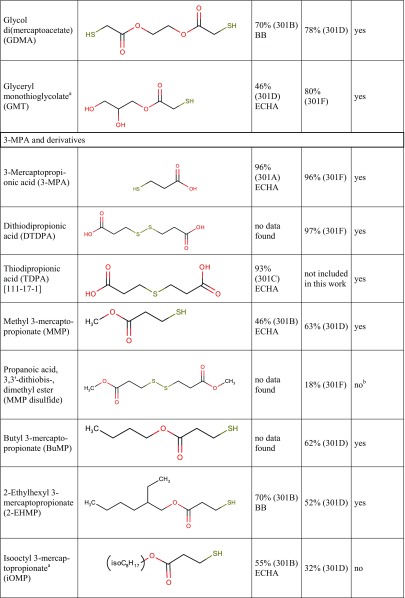

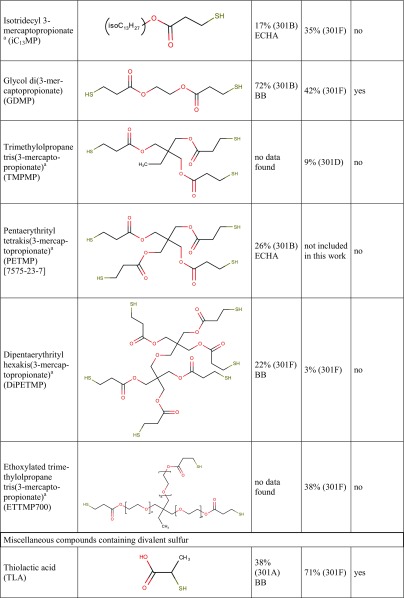

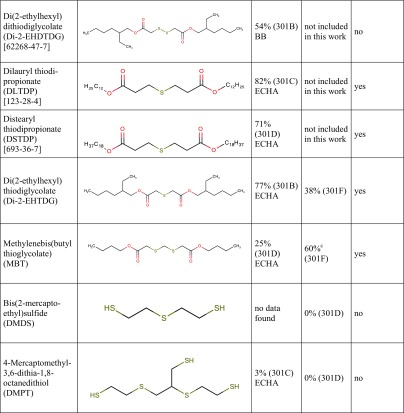
Synopsis of biodegradation results from the literature (ECHA or BB, i.e. ECHA database or original study reports provided by Bruno Bock) and from the present study

^a^Multiconstituent substance (MCS), for details see “Experimental”

^b^However, in one MRT experiment of MMP disulfide, both bottles reached the pass level of 60% (62 and 84%), thus failing with respect to criterion 1

^c^Mean value of 56.6 and 63.1% is 59.85%, just below 60% unless rounded

Column 3 shows biodegradation data found in the scientific or regulatory literature (e.g. ECHA database or unpublished original study reports). In order to use only reliable literature data, the quality of study results was assessed on a case-by-case basis using the Klimisch scoring system (Klimisch et al. 1997). Studies with a Klimisch score 1 (reliable without restriction) or 2 (reliable with restriction) only were considered for column 3. For example, among many results available in the literature for TGA (ranging from 0 to 100%), the reliable value of 67% (OECD 301D) was chosen from a Klimisch 2 study (van Ginkel and Stroo 1992).

Column 4 shows the highest experimental result for each substance obtained in the present OECD 301D or 301F studies (Klimisch 2), taken from Table 3.

The last column of Table 7 reports a consolidated assessment of all available ready biodegradability test results for each substance. This overall result is given as readily biodegradable (yes) or not readily biodegradable (no). The procedure leading to column 7 entries is based on the relevant guidelines saying

“Realising that ready biodegradability tests may sometime fail because of the stringent test conditions, positive test results should generally supersede negative test results” (ECHA 2017, page 208; OECD 2006, page 3), and

“When contradictory results in ready biodegradability tests are obtained the positive results could be considered valid irrespective of negative results, when the scientific quality of the former is good and the positive test results are well documented, …” (ECHA 2017, page 230).

We are aware that these formulations seem to be open to misuse by multiple testing and selective reporting, and this is another reason for showing in Table 3 all our experimental results.

Results in Table 7 can be summarised as follows:

1. Simple mercaptocarboxylic acids such as TGA, 3-MPA and TLA and their simple esters are readily biodegradable.
2. Those esters of the same acids that are not readily biodegradable are at the same time structurally complex (esters of branched higher or multifunctional alcohols) and multiconstituent substances. They nevertheless undergo considerable biodegradation in OECD 301 tests, so there is no reason to consider them persistent in the environment.
3. A disulfide motif does not prevent good biodegradability (DTDGA, DADTDG, DTDPA, MMP disulfide, Di-2-EHDTDG), nor does a sulfide (thioether) (TDPA, DLTDP, DSTDP, Di-2-EHTDG, MBT).
4. Di- or polymercaptans (without a favourable substructure) are not biodegradable (DMDS, DMPT) in OECD 301D or 301F tests.

These observations are in good agreement with the results shown in Table 2 for mercaptans. That is, the detrimental influence of a mercapto group can be counterbalanced by a favourable substructure such as an acid or ester group.

For mercaptan and sulfide functional groups, this is just the opposite of what is known for their oxygen analogues, hydroxyl groups enhance biodegradation while ether groups are detrimental (Boethling et al. 2007).

The higher mercaptoesters that proved not readily biodegradable have in common a rather high molecular weight associated with low solubility in water, and some bear branched higher alkyl groups. Both these characteristics are known to be detrimental to biodegradation. Others present at the molecule’s surface the mercapto groups only, i.e. they can be subsumed under item 4, not providing a convenient point of attack for bacteria. Further, all these substances have in common their MCS property. For MCSs such as petroleum products or mixtures of homologous compounds, e.g. technical surfactants, the guidelines acknowledge the onset of biodegradation to be delayed and/or the biodegradation curve to be less steep in comparison with the single constituents. Accordingly, the 10-day window is not applied for such substances (ECHA 2017, page 210; OECD 2006, paragraphs 43 and 44). By this logic, we expect homologous mixtures also to achieve less biodegradation than the single constituents after 28 days. The bacterial adaptation to many isomers, homologues or byproducts in low concentration may be slower than adaptation to a single chemical in higher concentration, since different bacterial strains or different degradative enzymes may be required. This may also apply to our MCSs that, though not homologous series, are complex mixtures of several related individual compounds.

With respect to inherent biodegradability tests (OECD 302A-C), the guidelines say

“Biodegradation above 20% of theoretical […] may be regarded as evidence of inherent, primary biodegradability” (OECD 2006, paragraph 36; ECHA 2017, page 216).

As far as substances measured here achieved above 20% biodegradation even in an OECD 301 test, *a fortiori* we consider this as evidence of their inherent primary biodegradability. Similarly,

“When results of ready biodegradability tests indicate that the pass level criterion is almost fulfilled (i.e. ThOD […] slightly below 60% […]) such results can be used to indicate inherent biodegradability” (OECD 2006) or “… can be used as evidence for inherent biodegradability” (ECHA 2017).

By these lines of argumentation, the mercaptoesters tested here, even those not formally readily biodegradable, can preliminarily be considered inherently biodegradable.

Interestingly, 3-MPA was enzymatically oxidised to the corresponding sulfinic acid by a special bacterial mutant (Bruland et al. 2009) and seems to be an intermediate in the metabolism of organic sulfur compounds (methionine, homocysteine) in natural environments (Salgado et al. 2015; references cited therein).

Recently, a multilinear model for description and prediction of biodegradation for general chemicals was proposed that uses additive functional group increments and is based on biodegradation data from several tests. Among all functional groups considered there, the mercaptan group turned out the strongest hindering biodegradation, while carboxylic acid and ester groups were found the most favourable (Vorberg and Tetko 2014). Our results are completely in line with this picture.

### Primary elimination and transformation products

Along with biodegradation by microorganisms, abiotic processes may occur in the environment or in biodegradation tests, e.g. photodegradation, hydrolysis, abiotic oxidation. Thus, aerobic disulfide formation from mercaptans is well known both during OECD 301 tests (footnote a in Table 2) and by oxidation in air-saturated tap water (TGA, OECD 2009; 3-MPA, unpublished, BB). Likewise, hydrolysis of esters during biodegradation tests is known (“Carboxylic acids and their esters”). With compound 2-EHTG, both these abiotic reaction types were observed in the 301C test.

In our experiments, based on HPLC-UV-MS/MS monitoring, rapid disulfide formation on test day 0 was observed for 3-MPA (→ DTDPA), MMP (→ MMP disulfide) and BuMP (→ BuMP disulfide), accompanied in the ester cases (MMP, BuMP) by formation of the corresponding monoester of DTDPA and of DTDPA itself during the test, for details, see the “Electronic supplementary material”.

The dimercapto thioether DMDS was quickly and completely transformed in CBT and MRT at day 0 to the cyclic disulfide 1,2,5-trithiepane. This disulfide, in contrast to the disulfides mentioned, was not further biodegraded up to day 28 in CBT or MRT, as it does not contain an ester or acid substructure. A corresponding oxidative cyclisation was observed for GDMP, for details, see the “Electronic supplementary material”.

Oxidative cyclisation of linear dimercaptans to cyclic disulfides was seen earlier under similar conditions (Houk and Whitesides 1987; Adamczyk et al. 2015). Formation of disulfides via oxidation of SH groups can be achieved using a wide variety of oxidants, among them molecular oxygen (Ozen and Aydin 2006; Carril et al. 2007; García Ruano et al. 2008; Abaee et al. 2011; Dewan et al. 2012; Shard et al. 2014), preferably under slightly alkaline conditions, in what is presumably a dimerisation of thiyl radicals formed by oxidation of thiolate anions (RSH → RS^−^ → RS·,2 RS· → RSSR). The transformation mercaptan → disulfide consumes an amount of oxygen (4 RSH + O_2_ → 2 RSSR + 2 H_2_O) that is small in most cases, e.g. complete conversion 3-MPA → 3-MPA disulfide requires 5% of the ThOD of 3-MPA. In our examples, this step was often so rapid as to occur during day 0 already to a large extent, in such a case the corresponding oxygen consumption will not even be measured in CBT or MRT since it happens before the bottles are closed.

The disulfide functionality, C-S-S-C, is essential in biochemistry, e.g. contributing to the conformational integrity of proteins including highly unusual natural products (Rücker and Meringer 2002) and in detoxification of xenobiotics via glutathione.

## Conclusion

New findings from this study are the following:

1. The rule of thumb for biodegradation of carboxylic acids/esters is well based on experimental evidence: such a functional group enhances biodegradation compared to a methyl group or to an H atom.
2. A rule of thumb for biodegradation of mercaptans can tentatively be established based on experimental evidence: a mercapto group hinders biodegradation compared to an H atom and even more so compared to a hydroxy group.
3. If both kinds of functional groups are present, then an intermediate behaviour results that strongly depends on the compound’s other structural characteristics. Simple mercaptocarboxylic acids and their esters are either readily biodegradable or at least biodegradable to some extent, i.e. they are not persistent compounds.

Biodegradation even of rather simple organic compounds to CO_2_, H_2_O, etc. is a multistep process, each step depending on catabolic capabilities (enzymes) of various microorganisms present. In standardised laboratory biodegradation tests as defined in the OECD 301 series, some influencing factors are not and cannot be standardised, most importantly the inoculum. Thus, in biodegradation experiments bacterial populations are used that differ with respect to the species and number of bacteria capable of degrading a particular compound. Necessary enzymes of more or less substrate selectivity may or may not be present or inducible. Appearance of bacterial strains actually degrading the test compound may require some time (lag phase) that depends on the particular course of expression and biosynthesis of enzymes, growth of bacteria and microevolution in the test flask. A conventional threshold for ready biodegradability such as 60% O_2_ consumption after 28 days may be passed in one experiment but not in another under seemingly identical conditions. For the mercaptans and mercaptoacids/esters considered here, concurrent abiotic reactions such as disulfide formation and ester hydrolysis complicate the picture. For these reasons, test results are often not reproducible. Therefore, conclusions on a particular compound’s biodegradability cannot be based on a single experiment. However, a consolidated assessment of all available biodegradation information for a compound class may lead to a coherent picture.

## Electronic supplementary material

ESM 1 (PDF 371kb)

## Footnotes

1 The 20% difference is usually interpreted as a 20 %points difference, since the unit of biodegradation is % (of a theoretical value). For example, if two replicate bottles show 41 and 59% biodegradation, the difference of 18 %points is considered still acceptable, though 59 and 41 (in any unit) differ by ~ 44 or ~ 30.5%, based on the lower or the higher value, respectively, which would be unacceptable when taking the criterion literally.
