# Supplementary material for: Biodegradation tests of mercaptocarboxylic acids, their esters, related divalent sulfur compounds and mercaptans
Source: Environ Sci Pollut Res Int. 2018 Apr 17;25(19):18393–411. doi: 10.1007/s11356-018-1812-x (PMC6061509; doi:10.1007/s11356-018-1812-x)
Supplement: Supplementary file 1 — (PDF 371kb) [file 11356_2018_1812_MOESM1_ESM.pdf]

SUPPLEMENTARY MATERIAL

Biodegradation tests of mercaptocarboxylic acids, their esters, related divalent sulfur compounds, and mercaptans

Christoph Rücker*[1], Waleed M. M. Mahmoud[1,2], Dirk Schwartz[3], Klaus Kümmerer[1]

*Corresponding author: e-mail: ruecker@leuphana.de, Telephone: +49 (0)4131 677 2834, Fax: +49 (0)4131 677 2848

[1]Institute of Sustainable and Environmental Chemistry, Leuphana University Lüneburg, Universitätsallee 1, D-21335 Lüneburg, Germany

[2]Analytical Chemistry Department, Faculty of Pharmacy, Suez Canal University, Ismailia 41522, Egypt

[3]Bruno Bock Thiochemicals, D-21436 Marschacht, Germany

Table S1. Test system of the aerobic ready biodegradation tests, for CBT columns 1-4, for MRT columns 1-5, “x” = added, “-” = not added

	1	2	3	4	5
	Inoculum blank	Quality control	Test proper	Toxicity control	Sterile control
Mineral medium	x	x	x	x	x
Inoculum	x	x	x	x	-
Test substance	-	-	x	x	x
Sodium acetate	-	x	-	x	-
Sodium azide	-	-	-	-	x

Experimental details of biodegradation experiments of several parent compounds

3-Mercaptopropionic acid (3-MPA) was completely transformed in both CBT and MRT at day 0 already into transformation product 3-MPA_TP1 that proved to be the corresponding disulfide (3-MPA disulfide = DTDPA) by retention time and MS comparison with authentic material (Table S2). Further, the day 0 transformed 3-MPA samples and authentic 3-MPA disulfide behaved identically in biodegradation tests: The disulfide was not further transformed in CBT or in the sterile bottle of MRT, however, at day 28 it had completely disappeared in the test and toxicity control bottles of MRT. No further products were found. These results are in concordance with the low biodegradation in CBT and high biodegradation in MRT observed for 3-MPA.

Methyl 3-mercaptopropionate (MMP) was transformed at day 0 of CBT already into three compounds, MMP_TP1 – MMP_TP3. The major product, MMP_TP1, was identified as the corresponding disulfide by retention time and MS comparison with authentic material. The minor product MMP_TP2 was 3-MPA disulfide (comparison with authentic material), while minor product MMP_TP3 was the monomethyl ester of 3-MPA disulfide according to MS data (Table S2). At CBT day 28 MMP disulfide had completely disappeared, while 3-MPA disulfide and the disulfide monoester were still present.

In MRT at day 0 the same three products resulted from MMP, at day 28 MMP disulfide had decreased, the disulfide monoester had increased with respect to day 0, 3-MPA disulfide was still present, and another minor peak (MMP_TP4) was observed but could not be identified.

Authentic MMP disulfide also was hydrolysed in CBT into its monoester (MMP_TP3) and 3-MPA disulfide (MMP_TP2). In MRT of MMP disulfide, the monoester was found, but no 3-MPA disulfide. Since the latter is completely degraded in MRT, we assume it was formed here also but was rather quickly biodegraded.

In conclusion, MMP was quickly transformed into its disulfide by oxidation while concomitant ester hydrolysis occurred in concurrence to biodegradation.

Butyl 3-mercaptopropionate (BuMP, t_R 12.4 min, Figure S1) on day 0 of CBT and of MRT was quickly but not yet completely transformed into BuMP_TP1 (t_R 17.6 min) and slowly into BuMP_TP2 (t_R 11.9 min), which were identified as the corresponding disulfide (BuMP disulfide) and the disulfide monobutyl ester, respectively, via MS (Table S2).

In CBT all three compounds had completely disappeared at day 28. In MRT on day 28 BuMP and BuMP disulfide had completely disappeared (Figure S1), while the disulfide monoester and another unidentified minor peak (BuMP_TP3, t_R 11.2 min) were still present. 3-MPA disulfide (BuMP_TP4, t_R 3.8 min) was found as a very small peak in BuMP MRT day 0 and day 28 samples, while 3-MPA was not detected. Another peak (BuMP_TP5, t_R 14.3 min) was observed but could not be identified.

Glycol bis(3-mercaptopropionate) (GDMP, t_R 10.7 min) also was eliminated from biodegradation test solutions from day 0 on, at day 28 the elimination was complete in both CBT and MRT. The major product (GDMP_TP1, t_R 9.6 min) is regarded as the corresponding cyclic disulfide, the product of oxidative cyclization, based on several major MS signals that are less heavy by 2 Dalton than those of GDMP (Table S2). This identification is supported by the retention time difference that is analogous to that observed between DMDS and its cyclic disulfide. Several minor peaks were found at day 28, but since some of these appeared already in the freshly prepared

test solution (day 0), they may be impurities rather than degradation products, and none of these was identified. Biodegradation of GDMP was low in CBT and intermediate in MRT in our experiments.

The dimercapto thioether DMDS (t_R 15.9 min) was quickly and completely transformed in CBT and MRT at day 0 to a product (DMDS_TP1, t_R 14.1 min) that was identified as 1,2,5-trithiepane by HPLC-UV comparison with authentic material (Table S2), a cyclic disulfide formed by oxidative cyclization. 1,2,5-Trithiepane was not further biotransformed up to day 28 in CBT or MRT.

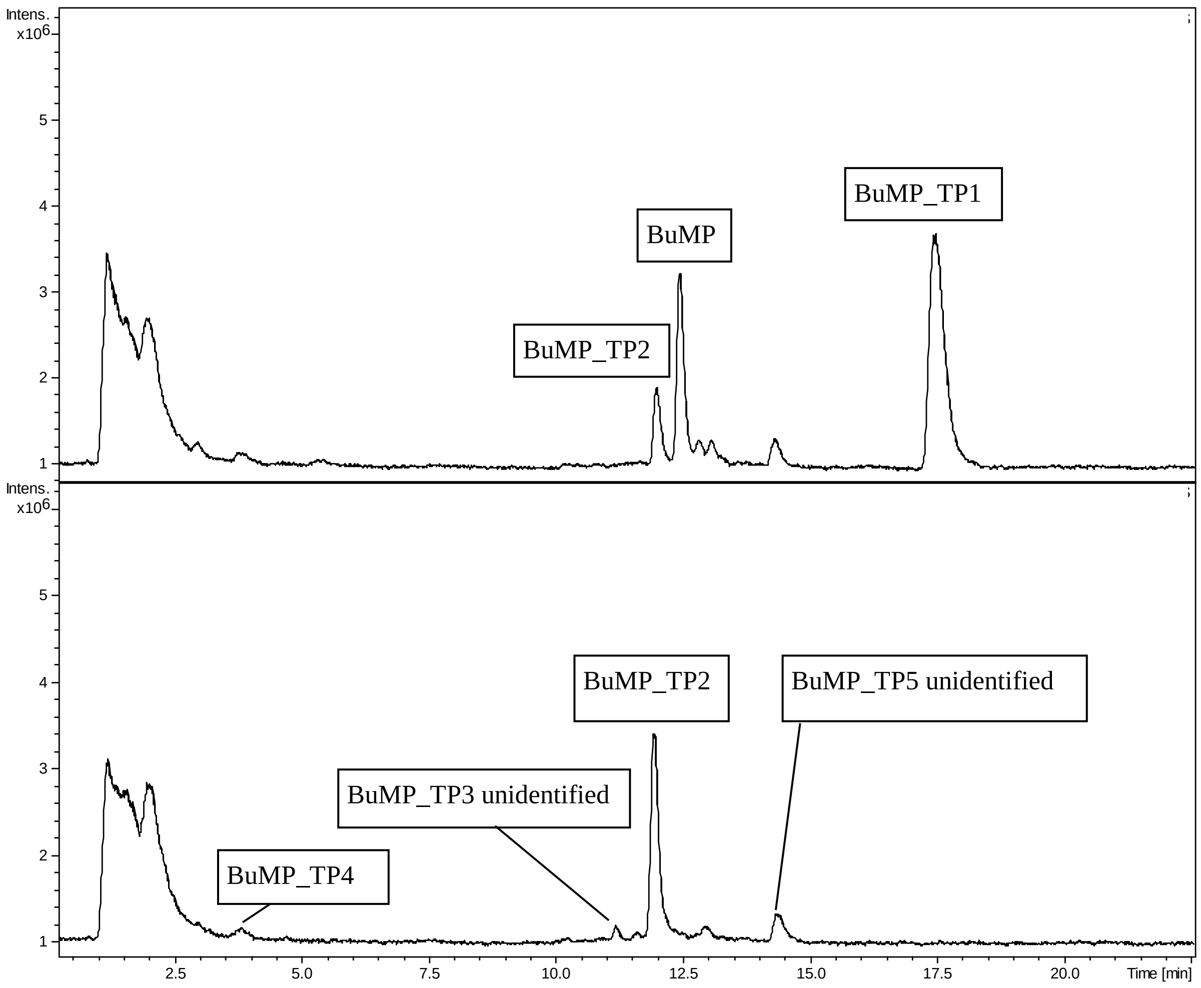

Fig. S1 Total ion chromatograms of BuMP samples in MRT taken at day 0 (top) and day 28 (bottom).

Table S2. HPLC retention times (t_R) and MS and MS^2 data of parent compounds and transformation products

**Compound**	**t_R [min]**	**Elution regime**	**MS m/z (intensity, explanation)**	**MS^2 *m/z* (intensity)**
3-MPA	2.5 / 3.9	I/II	-----	----
3-MPA_TP1	3.9	I	232.9 (100.0), 121.0 (96.8), 192.9 (81.8), 248.9 (40.6, M+K), 229.9 (28.4), 266.9 (17.9), 119.0 (17.6), 460.8 (15.2), 459.8 (13.6), 136.9 (13.0), 227.9 (12.6), 234.9 (10.8), 194.9 (10.7), 210.9 (10.7).	
authentic 3-MPA disulfide	3.9	I	121.0 (100.0), 232.9 (92.9, M+Na), 192.9 (75.6, M-H_2O+1), 210.9 (15.4), 227.9 (15.3), 119.0 (12.0), 164.9 (11.9), 136.9 (11.3).	192.9 → 121.0 (100.0), 174.9 (10.9)
MMP	5.5 / 7.3	I/III	-----	----
MMP_TP1	11.6	I	206.9 (100.0), 238.9 (44.8), 260.9 (30.0, M+Na), 174.9 (10.5)	
authentic MMP disulfide	11.6	I	206.9 (100.0, M-MeOH+1), 238.9 (26.6, M+1),174.9 (15.2, M-2MeOH+1), 208.9 (10.0)	238.9 → 206.9 (100.0), 207.9 (15) 206.9→174.9 (100.0), 178.9 (50.2), 119.1 (31.9), 135.0 (31.4), 175.9 (25.9), 150.9 (19.1), 87.3 (15.5), 137.0 (12.9), 121.0 (11.2), 176.9 (10.0)
MMP_TP2	3.8	I	232.9 (26.0), 172.0 (20.2), 121.0 (17.3), 192.9 (15.6)	
authentic 3-MPA disulfide	3.8	I	121.0 (100.0), 232.9 (90.5, M+Na), 192.9 (84.7, M-H_2O+1), 210.9 (15.0), 164.9 (14.9), 227.9 (14.0), 119.0 (13.9), 136.9 (11.8), 122.9 (10.3)	
MMP_TP3	9.2	I	206.9 (100.0, M-H_2O+1), 246.9 (57.7, M+Na), 224.9 (22.4, M+1), 174.9 (16.3, M-H_2O-MeOH+1), 208.9(10.6)	246.9 → 174.8 (100.0), 142.9 (85.7), 127.0 (39.2), 219.1 (32.4) 206.9 → 174.8 (100.0), 134.9 (55.6), 118.9 (33.6), 87.2 (22.6), 178.8 (20.5), 120.9 (11.7), 150.9 (11.4), 176.8 (10.9)

MMP_TP4 (uni-dentified)	9.6	I	228.9 (68.5), 206.9 (22.2), 142.9 (21.8), 174.9 (18.9), 246.9 (13.5)	228.9 → 168.0 (100.0), 208.8 (87.9), 244.8 (57.8) 207.0 → 174.9 (100.0), 142.9 (63.2), 192.9 (23.0)
BuMP	12.4	IV	163.0 (100.0, M+1), 107.1 (20.8), 184.9 (12.1, M+Na)	163.0→ 107.0 (100.0), 144.9 (66.5)
BuMP_TP1	17.6	IV	248.9 (100.0, M-BuOH+1), 323.0 (70.1, M+1), 345.0 (16.2, M+Na), 249.9 (13.1), 324.0 (11.5), 250.9 (10.4)	248.9 →161.0 (100.0), 121.0 (60.8), 192.9 (58.6), 174.9 (56.8), 105.1 (39.1), 176.9 (15.9), 129.1 (13.6) 323.0→ 248.9 (100.0), 161.0 (10.1)
BuMP_TP2	11.9	IV	248.9 (100.0, M-H_2O+1), 266.9 (34.4, M+1), 192.9 (27.8, M-BuOH+1), 161.0 (24.9, M-$C_3H_5O_2S$), 288.9 (15.3), 83.2 (14.9), 203.0 (14.6), 249.9 (11.9)	248 → 161.0 (100.0), 174.8 (61.0), 192.8 (55.9), 121.0 (55.3), 105.1 (41.2), 176.9 (17.1), 73.3 (12.4), 129.0 (12.0)
BuMP_TP3 (unidentified)	11.2	IV	234.9 (75.6), 216.9 (34.5), 256.9 (17.9), 142.9 (15.2), 163.0 (14.6), 235.9 (12.2)	234.9→ 216.9 (100.0),143.0 (70.4), 217.9 (12.7)
BuMP_TP4	3.8	IV	192.8 (80.8), 121.0 (30.0), 232.8 (19.1), 209.9 (19.1), 248.8 (17.3), 193.9 (13.9).	
BuMP_TP5 (uni-dentified)	14.3	IV	200.1 (100.0), 201.0 (13.4)	-----
DADTG	3.7	V	136.9 (100.0), 182.8 (70.1), 164.8 (54.9), 204.8 (51.3), 109.0 (37.7), 107.0 (16.9), 79.2 (12.0), 138.9 (10.6)	136.9 → 107.0 (100.0), 109.0 (94.9), 79.0 (24.6), 111.0 (15.4) 182.9 → 164.8 (100.0), 136.9 (57.0), 166.8 (16.0), 138.9 (15.7)
MBT	15.1	VI	309.0 (100.0), 161.0 (18.3), 331.0 (18.2), 310.0 (16.5), 326.0 (12.4), 311.0 (11.1)	-----
DMDS	15.92	VII	-----	-----
DMDS_TP1	14.13	VII	-----	-----
authentic 1,2,5-trithiepane	14.06	VII	------	----

GMDP	10.7	IV	255.9 (100.0), 260.9 (79.9, M+Na), 133.0 (74.9), 220.9 (15.0), 256.9 (12.2), 257.9 (10.8), 238.9 (10.7, M+1)	255.9 → 133.0 (100.0), 238.9 (19.4)<br>133.0 → 99.1
GDMP_TP1	9.6	IV	236.9 (100.0, M+1), 253.9 (79.2), 258.8 (36.4, M+Na), 238.9 (10.6), 237.9 (10.3)	253.9→ 236.9 (100)<br>236.9 → 131.0 (100.0)

Elution regimes for various parent compounds and their transformation products

Elution regime I: Gradient elution (solution A and solution B), 0 min 20% B, 2 min 20% B, 7 min 60% B, 16 min 60% B, 19 min 20% B, 25 min 20% B.

Elution regime II: Isocratic elution: eluent mixture of solution A and solution B (90:10 v/v).

Elution regime III: Isocratic elution: eluent mixture of solution A and solution B (85:15 v/v).

Elution Regime IV: Gradient elution (solution A and solution B) 0 min 20% B, 2 min 20% B, 7 min 70% B, 20 min 70% B, 23 min 20% B, 30 min 20% B.

Elution Regime V: Isocratic elution, solution A and solution B 85:15 (v/v).

Elution Regime VI: Gradient elution (solution A and solution B) 0 min 30% B, 2 min 30% B, 7 min 70% B, 16 min 70% B, 19 min 30% B, 25 min 30% B.

Elution Regime VII: Isocratic method: eluent mixture of solution A and solution B (70:30 v/v).

MS operation parameters used in MS^n of organosulfur compounds

General MS operating parameters: Maximum accumulation time 200 ms, dry gas temperature 350°C, dry gas flow 12 l/min, and nebulizer pressure 30 psi.

For 3-MPA, 3-MPA disulfide, MMP, MMP disulfide, MBT, and their transformation products:
Scan range 50 m/z to 500 m/z, HV end plate offset -500 V, capillary voltage -3900 V, trap drive 36.9,
lens 1 -5 V, lens 2 -60 V, capillary exit 108.1 V, skimmer 40 V, octopole 1 12 V, octopole 2 1.7 V, and octopole reference amplitude 137.7 Vpp.

For BuMP and its transformation products:
Scan range 50 m/z to 400 m/z, HV end plate offset -500 V, capillary voltage -4500 V, trap drive 32.4,
lens 1 -3.5 V, lens 2 -55 V, capillary exit 50 V, skimmer 32 V, octopole 1 12.69 V, octopole 2 5.56 V, and octopole reference amplitude 50 Vpp.

For GDMP and its transformation products:
Scan range 50 m/z to 500 m/z, HV end plate offset -500 V, capillary voltage -3600 V, trap drive 39.2,
lens 1 -3.3 V, lens 2 -61 V, capillary exit 50 V, skimmer 33.4 V, octopole 1 11.02 V, octopole 2 3.9 V, and octopole reference amplitude 112.5 Vpp.

For DADTG:

Scan range 50 m/z to 400 m/z, HV end plate offset -500 V, capillary voltage -4200 V, trap drive 32, lens 1 -5.5 V, lens 2 -100 V, capillary exit 50 V, skimmer 34.8 V, octopole 1 14.83 V, octopole 2 3.67 V, and octopole reference amplitude 79.2 Vpp.
